# The Trends and Hotspots in Premature Ovarian Insufficiency Therapy from 2000 to 2022

**DOI:** 10.3390/ijerph191811728

**Published:** 2022-09-17

**Authors:** Yan Tong, Nan Cheng, Xinran Jiang, Kai Wang, Fei Wang, Xinxin Lin, Fang Wang

**Affiliations:** 1 Department of Literature and Information of Library, Shenyang Pharmaceutical University, Shenyang 110016, China; 2 School of Life Science and Biopharmaceutics, Shenyang Pharmaceutical University, Shenyang 110016, China; 3 School of Pharmacy, Shenyang Pharmaceutical University, Shenyang 110016, China; 4 School of Functional Food and Wine, Shenyang Pharmaceutical University, Shenyang 110016, China

**Keywords:** primary ovarian insufficiency, therapy, bibliometric analysis, science mapping

## Abstract

This study aims to map the knowledge structure and themes trends of primary ovarian insufficiency (POI) therapy to help researchers rapidly master the hotspots and prospects of POI therapy from the increasing number of publications. The literature search and bibliometric analyses were performed by using Web of Science Core Collection and VOSviewer. Annual publications from 2000 to 2022 continued to increase with some fluctuations. The most productive country, organization, and journal were the USA, Shanghai Jiao Tong University, and *Human Reproduction*, respectively. Harvard University was the organization with the highest citation. *Fertility and Sterility* and Nelson, L.M. were the most influential journal and author, respectively. Seven clusters separated by keywords association showed the extensive scope of POI therapy. The hotspots of POI therapy were hormone replacement therapy and fertility preservation, and the innovative treatment strategies including in vitro activation and mesenchymal stem cells had development potential. In addition, our result showed that the high-cited articles were published in journals with high impact factors. The paper provides a comprehensive overview of the development and hotspots of POI therapy, allowing researchers to recognize the current status and future directions of POI therapy.

## 1. Introduction

Premature ovarian insufficiency (POI) is a clinical syndrome defined by the premature loss of ovarian activity, leading to a chronic hypo-estrogenic state in women under the age of 40 years [1]. POI can be triggered by a multitude of exogenous factors through several mechanisms [2,3]. Menstrual disturbances, raised gonadotropin levels, and sex-steroid deficiency are the core characteristics and can result in long-term physical and psychological consequences in POI patients [1]. It is well known that POI is associated with a significant increase in the risk of osteoporosis [4], cardiovascular disease [5], and neurological disorders [6], while one of the most distressing effects of POI is the associated subfertility [7]. In the earlier stages of the POI process, POI therapy can prevent deterioration in the quality of life, reduce the risk of long-term sequelae, and improve fertility [8].

A considerable amount of research related to POI therapy was published and this topic has gained increasing attention among scholars. It is widely accepted that the most prevalent POI therapy is hormone replacement therapy (HRT). Appropriate hormone replacement therapy may replace premenopausal levels of ovarian sex steroids and improve the quality of life for women with POI and ameliorate associated health risks [9]. It should be recommended to the patients without contraindications and continued at least until the average age of menopause. HRT can relieve symptoms and improve quality of life; however, it cannot restore ovarian functions, including secretion, ovulation, and fertility [8,10]. For fertility preservation, ovum donation in vitro fertilization remains an effective way to achieve a family in POI, but this method is not acceptable to a significant proportion of women for cultural reasons [8]. It is therefore imperative that research into novel approaches for fertility restoration continues in women with POI. Elective freezing of oocytes and ovarian tissue are tried and tested interventions that allow for a preemptive approach to fertility preservation in those deemed at risk for POI, such as POI patients with genetic predispositions, cancer survivors, or women with autoimmune conditions [11]. In the last thirty years, specialists in assisted reproduction have focused their attention on the final stages of folliculogenesis, those that depend on the action of gonadotropins. Recently, many potentially promising treatment modalities are already being explored, such as mesenchymal stem cells, in vitro activation, and platelet-rich plasma.

With the fast development of the internet and the arrival of the big data era, the rapid growth of the number of publications makes it difficult for researchers to quickly understand the status quo and trend of POI therapy. Therefore, it is necessary to conduct a multidimensional and quantitative analysis of the research status of POI therapy. Bibliometrics can quantitatively analyze the development trend of the subject and the rule of literature quantitative change. VOSviewer is a software tool for constructing and visualizing bibliometric networks [12,13]. These networks may include journals, researchers, or individual publications, citations, bibliographic coupling, co-citation, co-authorship relationships, or co-occurrence networks. The viewing capabilities of VOSviewer are more powerful than that of most computer programs that are used for bibliometric mapping [12,14]. VOSviewer has been used successfully in various subject areas such as energy [15], geosciences [16], medicine [17], and engineering [18]. 

In this study, we retrieved literature on therapy for POI from the Web of Science Core Collection and used VOSviewer to analyze the distribution of publication years, countries and regions, organizations, journals, core authors, keywords, and key references. The purpose of the study was to map the knowledge structure and themes trends of primary ovarian insufficiency (POI) therapy to help researchers rapidly master the hotspots and prospects of POI therapy from the increasing number of publications. Section 2 contains the data source, search strategy, and the methods of bibliometric analysis and network visualization. In Section 3, the results of the analysis are introduced. Section 4 and Section 5 present the discussion and conclusions, respectively.

## 2. Materials and Methods

### 2.1. Data Source and Search Strategy

The literature data for this bibliometrics study were retrieved from the Web of Science Core Collection. The Web of Science Core Collection can search the world’s leading scholarly journals, proceedings in the sciences, social sciences, and arts and humanities, and navigate the full citation network [19]. All cited references for all publications are fully indexed and searchable.

**Figure 1:**
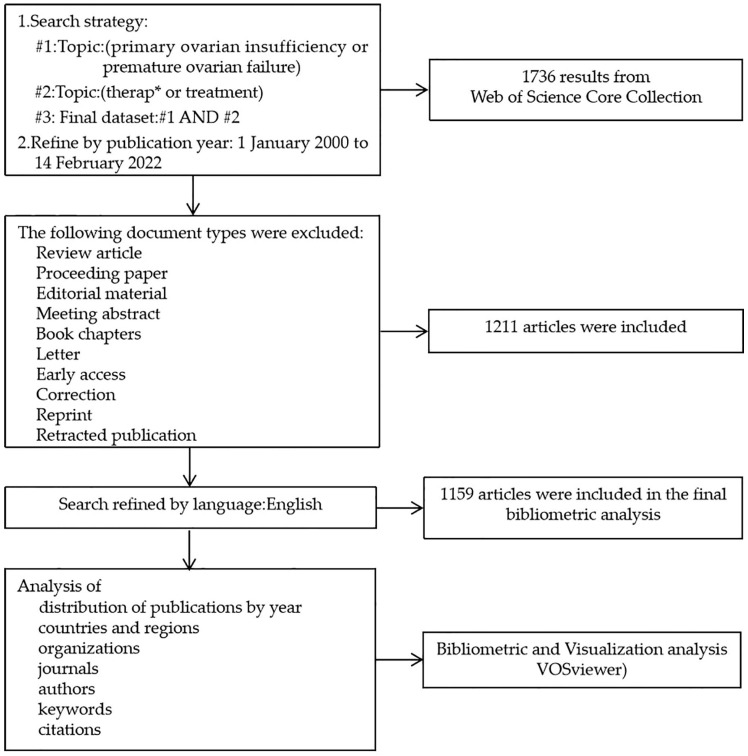
A frame flow diagram of the document search and selection process. The diagram shows detailed selection criteria for POI therapy publications from the Web of Science Core Collection database and the steps of the bibliometric analysis.

To perform a systematic analysis of the therapy for POI, we chose original articles and other types of documents (review article, proceeding paper, editorial material, meeting abstract, book chapters, letter, early access, correction, reprint, retracted publication) were not considered. The search strategy was as follows: TS = (primary ovarian insufficiency or premature ovarian failure) AND TS = (therap* or treatment). A total of 1159 documents from 1 January 2000 to 14 February 2022 were used for visualization analysis from the Web of Science Core Collection. The search and download of data were completed on 14 February 2022. Figure 1 presents the literature search and selection process.

### 2.2. Results Analysis

VOSviewer provides three visualizations, referred to as network visualization, overlay visualization, and density visualization. In network visualization, items are represented by a circle. The size of the circle of an item is determined by the weight of the item. The higher the weight of an item, the larger the circle of the item. The color of an item is determined by the cluster to which the item belongs. Lines between items represent links. The overlay visualization is identical to the network visualization except those items are colored differently. If items have scores, the color of an item is determined by the score of the item, whereby default colors range from blue (lowest score) to green to yellow (highest score). In the density visualization, each point in the density visualization has a color that indicates the density of items at that point. Colors range from blue to green to yellow. The larger the number of items in the neighborhood of a point and the higher the weights of the neighboring items, the closer the color of the point is to yellow. Furthermore, in order to allow the results to be reproduced, we will indicate all the parameters used for building the maps [20,21].

In this paper, the retrieval characteristics of therapy for POI included the distribution of publication years, countries and regions, organizations, journals, core authors, keywords, and key references. Bibliometric analysis and network visualization were performed with VOSviewer (Download VOSviewer 1.6.18 for Microsoft Windows systems, https://www.vosviewer.com/, accessed on 15 April 2022). The keywords and references were chosen to anticipate the research hotspots and prospects. Keywords and reference analyses were performed with VOSviewer [22,23].

## 3. Results

### 3.1. Distribution of Publications by Year

**Figure 2:**
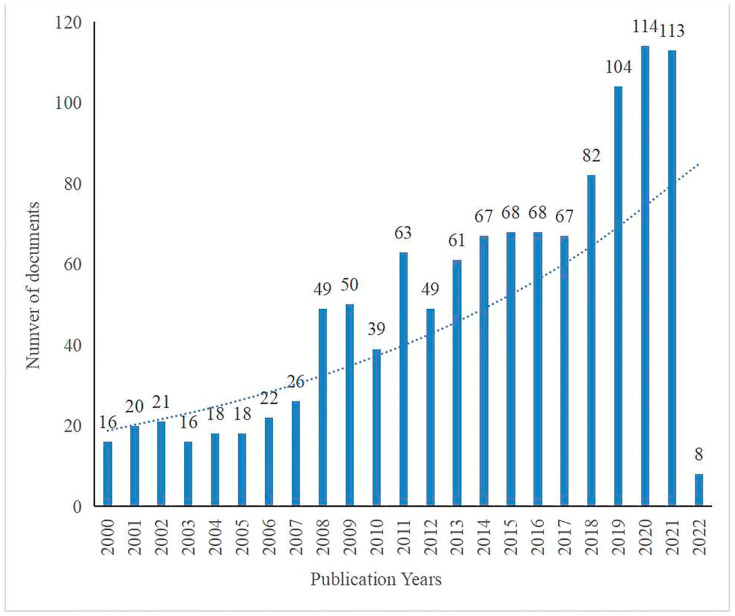
Distribution of publications by year on therapy for POI from 2000 to 2022. The bibliographic data were downloaded on 14 February 2022.

The chronological distribution of published documents on therapy for POI is shown in Figure 2. The line chart illustrates that the number of documents increased relatively slowly from 2000 (*n* = 16, 1.381%) to 2007 (*n* = 26, 2.243%), while the number of documents rose sharply from 2017 (*n* = 67, 5.781%) to 2021 (*n* = 113, 9.750%) and reached a peak in 2020. From the annual publications, therapy for POI acquired increasing attention, indicating that it gradually became a research hotspot, even into the future.

### 3.2. Countries and Regions

Statistical analysis showed that research teams from 66 countries and regions published 1159 articles. Many of these publications were produced in collaboration with multiple countries and regions. Among the top 10 countries with the largest number of publications, the USA had the largest number of publications (*n* = 297, 25.63%), followed by China (*n* = 230, 19.84%) and Italy (*n* = 84, 7.25%), as shown in Table 1.

### 3.3. Organizations

**Figure 3:**
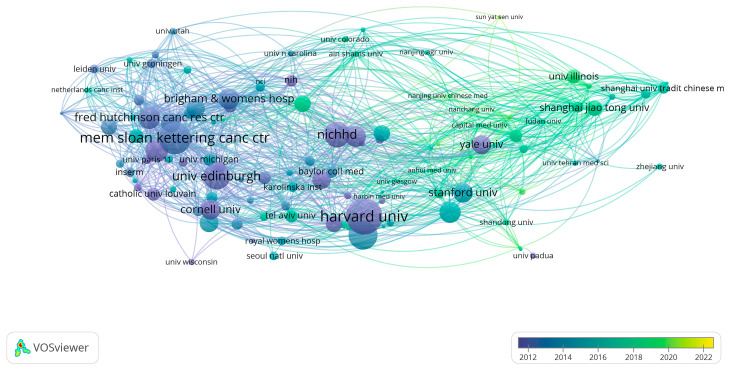
Organizations with citation relations shown as an overlay graph plotted with VOSviewer. The analysis method was Linlog/modularity. The weight was citations. The thickness of lines indicates the strength of the relationship. The color indicates the average published year.

VOSviewer analysis showed that 1703 organizations published 1159 documents, of which 114 publications met the statistical threshold (we ignored documents co-authored by a large number of organizations, the maximum number of organizations per document was 25, the minimum number of documents of an organization was 5, and the minimum number of citations of an organization was 1). After disjointed organizations were excluded from the statistical analysis, 113 organizations were used for visual analysis. The most productive organizations among the top 10 were Shanghai Jiao Tong Univ, followed by Nanjing Med Univ and Univ Estadual Campinas. There were three Chinese organizations in the top 10, but the total of citations of the three Chinese institutions (*n* = 1090) was lower than that of Harvard University (*n* = 2001), as shown in Table 2. In addition, the organizations with the most citations included Harvard University, Mem Sloan Kettering Canc Ctr, and Univ Edinburgh. Furthermore, the node in purple indicated that the organization’s average publishing year was 2012. Harvard University, which was represented by a purple node, had a higher number of citations than any other organization, suggesting that it might be a very important institution for the study of therapy for POI (Figure 3).

### 3.4. Journals

It is helpful to identify core journals by analyzing the distribution of the publication sources. Based on data analysis, the documents related to therapy for POI published from 2000 to 2022 were distributed in 466 different journals (of the 466 sources, 51 met the threshold). As shown in Table 3, the most prolific journals were *Human Reproduction* and *Fertility and Sterility*. The range of 2020 impact factors, given in Table 3, was 2.260 to 7.329. The 2020 impact factor of *Fertility and Sterility* was the highest, and that of *Gynecological Endocrinology* was the lowest. Judging from the number of publications and the impact factor of journals, *Fertility and Sterility* might be the most influential journal.

Journal co-citation analysis is a quantitative research method in bibliometrics and scientometrics. It has been widely used in many research fields by scholars. Through the analysis of journal co-citation, we can locate and classify journals, determine the core or edge position of journals in the discipline, and then evaluate academic journals [24].

**Figure 4:**
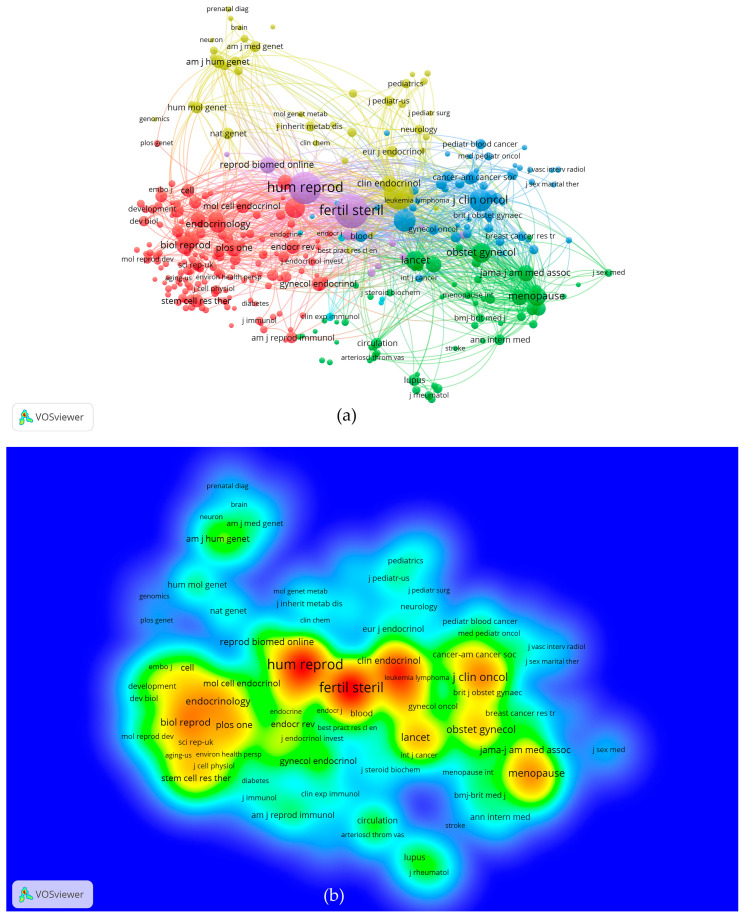
VOSviewer visualization map of most commonly cited journals related to therapy for POI. (**a**) Co-citation network of the most commonly cited journals. Of the 4802 cited journals, 331 met the threshold. (**b**) The density map of the most commonly cited journals. The big nodes represent journals with large numbers of citations.

The VOSviewer visualization map of most commonly cited journals related to therapy for POI showed that of the 4802 cited journals, 331 met the threshold (the minimum number of citations of a source was 20). Each point in the density visualization has a color that indicates the density of the journal. Colors range from blue to red. The larger the number of journals in the neighborhood of a point and the higher the weights of the neighboring journal, the closer the color of the point is to red. The big nodes represent journals with large numbers of citations. The top co-cited journals were *Fertility and Sterility* (IF = 7.329), *Human Reproduction* (IF = 6.918), and *Journal of Clinical Endocrinology Metabolism* (IF = 5.958) (Figure 4a,b). These results indicate that the above journals have strong academic performance in the field of therapy for POI.

### 3.5. Authors

Core author analysis is helpful to explore the distribution of documents. The indicators for evaluating core authors include the number of published papers and total citations. Between 2000 and 2022, 6144 authors published 1159 papers on therapy for POI. The top 10 core authors who published papers on the therapy for POI were mainly from the USA, Japan, and Israel. Nelson, L.M. ranked first in the number of references (*n* = 18) and total citations (*n* = 1587), indicating that Nelson, L.M. was the most influential researcher on the therapy for POI, as shown in Table 4.

Scientific collaboration networks are a hallmark of contemporary academic research. Researchers are no longer independent players, but members of teams that bring together complementary skills and multidisciplinary approaches. Social network analysis and co-authorship networks are increasingly used as powerful tools to assess collaboration trends and to identify leading scientists and organizations.

**Figure 5:**
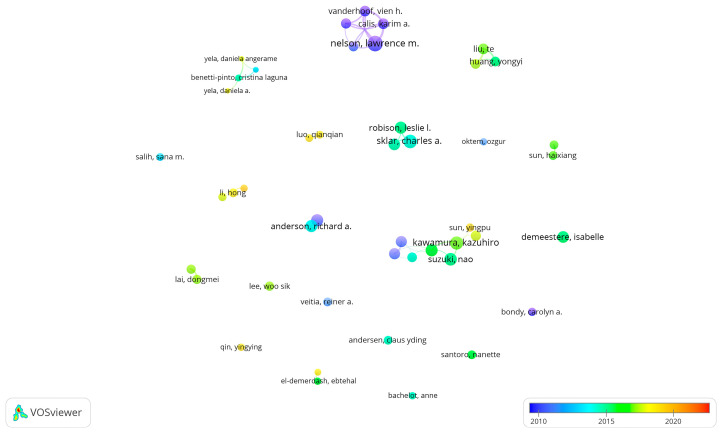
Overlay visualization of co-authorship analysis of authors. The analysis method was Linlog/modularity. The weight was citations. The thickness of the line indicates the strength of the relationship. The color of the circle represents the average published year.

Co-authorship of authors is shown in Figure 5. Of the 6144 authors, 46 authors were included in the statistical visualization analysis because they met the statistical threshold. Among the influential authors, Nelson, L.M. collaborated more closely with Vanderhoof, V.H. and Calis K.A. Worked closely with Troendle, J.F. In addition, the average year of publication of their papers was mainly in 2010, showing that they had paid attention to the therapy for POI research early. More and more new research teams join the research on the therapy for POI, indicating that therapy for POI has always been a research hotspot [25].

**Figure 6:**
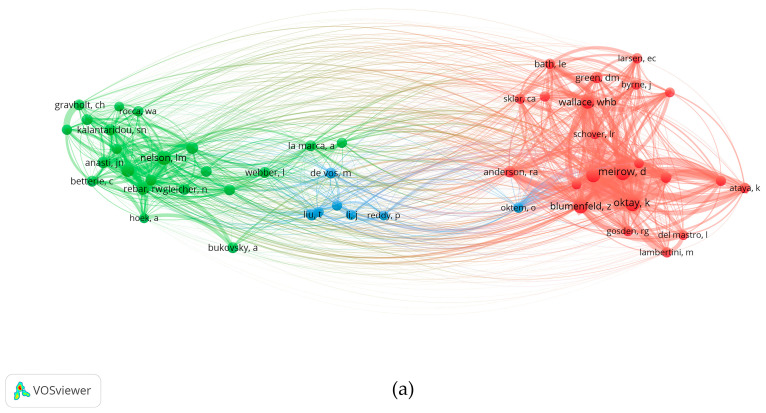

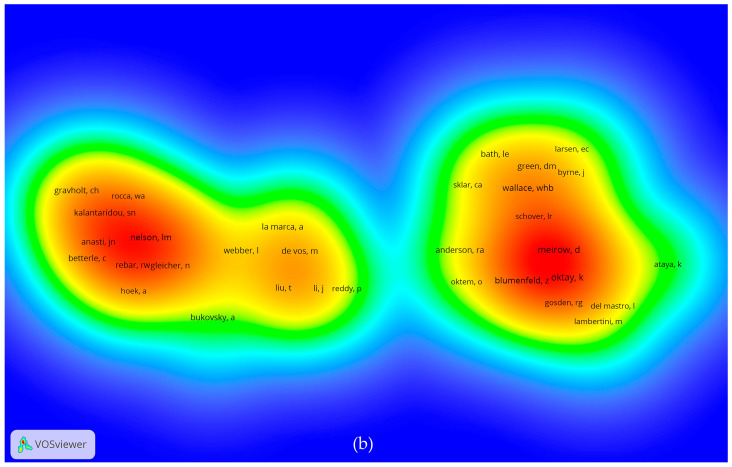
Author co-citation visualization map on therapy for POI. (**a**) The network map of co-citation (cited authors). The threshold for the minimum number of citations of an author was set to 50. A total of 51 authors who met the threshold were identified. (**b**) The density map of co-citations (cited authors). The size of a node is proportional to the total number of citations of the author, and the thickness of the connecting line indicates the strength of the co-citation link.

A co-citation (cited authors) visualization map was also generated using VOSviewer software [26]. The co-citation frequency of Meirow, D. and Oktay, K. was higher, it indicated that their academic relationship was closer and the “distance” was closer. Nelson, L.M. and Rebar, R.W. were another pair of authors with simultaneous high citations. Through the analysis of co-citation (cited authors), the core authors in the field of therapy for POI can be classified according to this “distance”. It can be seen that Meirow, D., Oktay, K., and Nelson, L.M. had made significant contributions to therapy for POI (Figure 6a,b).

### 3.6. Keywords

**Figure 7:**
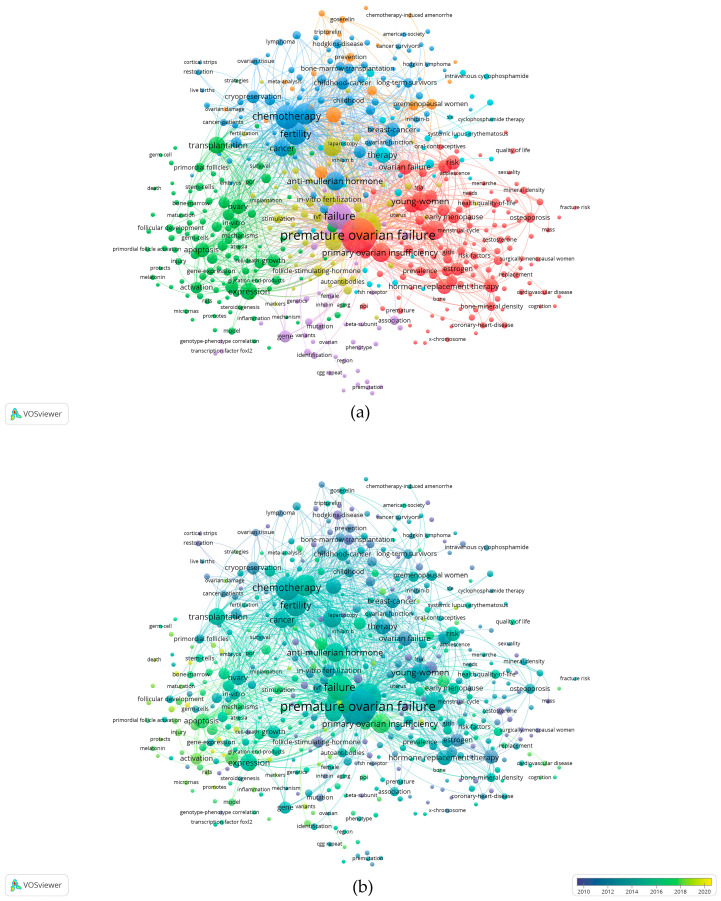

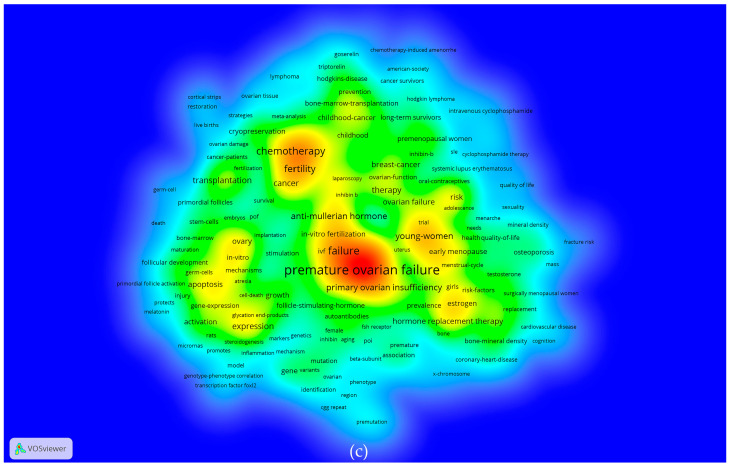
Co-occurrence analysis of keywords. (**a**) Network visualization mapping of the keywords. The nodes represent keywords and the size of the nodes is proportional to the frequency of the keywords. The nodes and lines of different colors symbolize diverse keyword clusters. (**b**) Overlay visualization mapping of the keywords. Distribution of keywords on the average appearance time. The color of each node corresponds to the average publication year. The size of a node is proportional to the frequency of occurrence of the keyword, and the thickness of the connecting line indicates the strength of the keywords’ co-occurrence link. (**c**) Density visualization mapping of the keywords. The closer the keyword node color is to red, the higher the frequency of its co-occurrence.

A total of 4555 keywords were retrieved from 1159 documents, and 477 met the threshold (the minimum number of occurrences of a keyword was 5 times). Seven research clusters were separated based on their association. The network visualization mapping of the keywords was shown in Figure 7a and the top five keywords in each cluster are listed in Table 5. The red cluster (cluster 1) was constituted mainly of “premature ovarian failure”, “hormone replacement therapy”, “menopause”, “primary ovarian insufficiency”, and “young-women”. The cluster mainly described the improvement effect of HRT on quality of life and associated health risks for women with POI. The green cluster (cluster 2) focused on “expression”, “transplantation”, “granulosa-cells”, “mice”, “apoptosis”, and “ovary”. It primarily introduced the pathogenesis and various novel therapeutic strategies of POI in this cluster. The dark blue cluster (cluster 3) primarily consisted of “chemotherapy”, “fertility”, “fertility preservation”, “anti-mullerian hormone”, and “cancer”. In this cluster, the various methods of fertility preservation in childhood and adolescent female tumor survivors were investigated. The yellow cluster (cluster 4) was constituted mainly of “women”, “pregnancy”, “infertility”, “in vitro fertilization”, and “follicle-stimulating-hormone”. This cluster mainly investigated the application and development of a series of assisted reproductive technologies for infertility in patients with POI. In the purple cluster (cluster 5), “failure”, “premature ovarian insufficiency”, “gene”, “association”, and “mutation” were the chief keywords. the study in this cluster focused on those genes involved in the etiology of POI and the genetic diagnosis. The light-blue cluster (cluster 6) was constituted mainly of “therapy”, “systemic-lupus-erythematosus”, “impact”, “gonadotoxicity”, and “follow-up”. It primarily investigated the relationship between POI and systemic lupus erythematosus. The risk of developing POI and gonadotoxicity of cyclophosphamide for the treatment of systemic lupus erythematosus were also discussed in this cluster. In addition, “cyclophosphamide”, “reserve”, “premenopausal women”, “breast cancer”, and “adjuvant chemotherapy” were the chief components of the orange cluster (cluster 7). The association between cyclophosphamide adjuvant chemotherapy and ovarian function in young women with breast cancer was investigated in this cluster.

Term maps were developed to depict relationships and occurrences of terms related to the search topics. To explore the changes in hotspots of POI over a period of time, overlay visualization mapping of the keywords was generated using VOSviewer software (Figure 7b). The timeline was divided into the following three stages and the top 10 keywords in each stage are listed in Table 5. In the primary stage (from 2000 to 2009), the top 10 keywords had fewer occurrences (ranging from 9 to 25) compared with those that appeared in the middle stage (from 2010 to 2017). This is the initial stage for the research studying POI and exploring HRT therapy. From 2010 to 2017, the number of total keywords increased. “Premature ovarian failure”, “women”, “failure”, “chemotherapy”, “fertility”, “fertility preservation”, “anti-mullerian hormone”, “hormone replacement therapy”, “menopause”, and “primary ovarian insufficiency” were the top 10 keywords with more occurrences (from 103 to 381) (Table 5, Figure 6b). The literature in the middle stage mainly focused on the method of fertility preservation, especially in patients with POI undergoing chemotherapy and the indications, safe and effective treatment options, and risks of HRT for patients with POI. In the last five years, the keywords related to the therapy for POI in the top 10 keywords were “mesenchymal stem-cells”, “acupuncture”, “antioxidant”, “in vitro activation”, and “platelet-rich plasma” (Table 5). Although the occurrences of the top 10 keywords were only from 7 to 69, these keywords indicated that these innovative treatment strategies have great therapeutic potential in clinical practice and will bring new hope to patients with POI.

According to the density map of keywords (Figure 7c), “premature ovarian failure”, “failure”, “primary ovarian insufficiency”, “anti-mullerian hormone”, “chemotherapy”, “fertility”, and “young-women” had higher weight.

### 3.7. Citations

According to the citation analysis of the articles, the top 10 cited articles are listed in Table 6. The range of the number of citations was from 344 to 1067. “Tailoring therapies-improving the management of early breast cancer: St Gallen international expert consensus on the primary therapy of early breast cancer” ranked first, which was published by Coates in 2015 and was cited 1067 times. This study endorsed the role of ovarian function suppression with either tamoxifen or exemestane for patients at higher risk and noted the value of an LHRH agonist given during chemotherapy for premenopausal women with ER-negative disease in protecting against premature ovarian failure and preserving fertility [27]. “Livebirth after orthotopic transplantation of cryopreserved ovarian tissue” ranked second and was published by Donnez in 2004. This article described a livebirth after orthotopic autotransplantation of cryopreserved ovarian tissue and suggested that cryopreservation of ovarian tissue should be offered to all young women diagnosed with cancer [28]. “Primary ovarian insufficiency” ranked third and was cited 595 times. This study introduced the cause, evaluation, and management of primary ovarian insufficiency [29]. The highest impact factor (IF) among the top 10 journals is 91.24 (*New England Journal of Medicine*) and 80% of journals were classified in Q1 according to the 2020–2021 journal citation reports (JCR).

**Figure 8:**
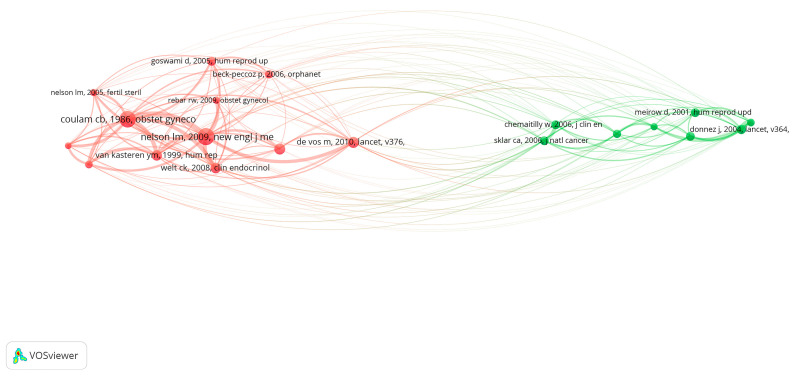
Co-citation analysis of references. The size of the node indicates the record of documents cited.

In addition to citation analysis, co-citation analysis is also one of the important methods to evaluate the core literature. The top 20 co-cited references were selected, and each of the 12 co-cited references was cited at least 32 times. Among the 20 references with high co-citation frequency, Nelson et al., published in the *New England Journal of Medicine* (2009), had the highest co-citation frequency (126 citations), followed by Coulam et al. in *Obstetrics and Gynecology* (1986, 121 citations), De Vos et al. in *Lancet* (2010, 69 citations), and Webber et al. in *Human Reproduction* (2016, 66 citations). The number of co-citations in the 16 references ranged from 32 to 64. References (12/12,747, 0.09%) with co-citations ≥ 102 (T = 102) were used to construct the co-citation map (Figure 8). “Nelson LM, *New England Journal of Medicine*, 2009” had the largest size and had active co-cited relationships with “Welt CK, *Clinical Endocrinology*, 2008” and “Rebar RW, *Obstetrics and Gynecology*, 2009” (Figure 8).

## 4. Discussion

In this study, we used bibliometrics to analyze the literature related to the therapy for POI. The results showed that in the past 20 years, the therapy literature on POI increased year by year, and the citation of literature also showed a significant growth trend (Figure 2).

An important indicator to measure the level of scientific research in a discipline of a country or an organization is the number of publications published. Data analysis showed that China and the United States were the two countries with the largest number of therapy papers published on POI (Table 1 and Table 2), indicating that they have a very important influence in the field of POI research. In addition, Shanghai Jiaotong University was the organization that published the most therapeutic papers on POI (the green node represents Shanghai Jiaotong University, and the green node indicates the organization’s average publication year was 2020), which meant it might be an emerging research organization (Figure 3).

Journal indicators obtained from the bibliometric analysis can provide a reliable reference for researchers to search documents or submit manuscripts. Our results showed that *Human Reproduction* published the highest number of papers about therapy for POI and had the highest number of total citations (Table 3). Our results also showed that the top three co-cited journals were *Fertility and Sterility*, *Human Reproduction*, and *Journal of Clinical Endocrinology Metabolism* (Figure 4a,b). These results suggest that these active journals can provide a reliable reference for scholars concerned with therapy for POI.

Based on the web of science database, Nelson, L.M. published the highest number of papers about the therapy for POI. (Table 4). Furthermore, Nelson, L.M. Vanderhoof, V.H., Calis K.A., Troendle, J.F., and Meirow, D. cooperated closely and published a considerable number of highly cited publications, as evidenced in the co-authorship network visualization map and co-citation visualization map (Figure 5 and Figure 6). Therefore, they can be regarded as leaders in therapy for POI.

Keywords can provide immediate information about the themes and the main theme in a particular study. In this study, seven research clusters were separated based on the association of keywords by keyword co-occurrence analysis. These clusters encompassed almost all aspects of POI therapy. As shown in Figure 7a, the high-frequency keywords were mainly “premature ovarian failure”, “failure”, “chemotherapy”, “fertility”, “anti-mullerian hormone”, “primary ovarian insufficiency”, “young-women”, and “hormone replacement therapy”, etc. Almost all of these keywords were located in the middle of the network as core words and they reflected the areas of interest in the field of therapy for POI. In this paper, the changes in relevant keywords at different stages were also analyzed. During the primary stage, the methods of maintenance of the POI patient’s well-being and the possible infertility therapeutic strategy began to be explored. The numbers of keywords and the occurrences of the top 10 keywords in the middle stage were more than those in the primary stage, indicating that therapeutic strategies of POI induced by various factors had attracted more attention. During the middle stage, the articles on POI therapy mainly focused on HRT and the method of fertility preservation, especially in patients with POI undergoing chemotherapy. HRT was thought to be a mainstay and effective approach to treating the symptoms of hypoestrogenism. The choice of HRT formulations should closely mimic normal ovarian steroid hormone production and provide sufficient levels of estradiol to reduce menopausal symptoms, maintain bone density, minimize the psychological impacts of estrogen deficiency, and protect against the early progression of cardiovascular disease and dementia. The studies during this stage suggested that appropriate HRT to replace premenopausal levels of ovarian sex steroids was paramount to increasing the quality of life for women with POI and ameliorating associated health risks [9,30]. Webber (2017) and Sassarini (2015) discussed the safe and effective HRT options and thought that patient preference for route and method of administration of each component of HRT must be considered when prescribing [30,31]. During this stage, several options have been proposed for preserving the fertility of women with POI induced by genetic defects, chemotherapy, radiotherapy, or surgery, including medical therapy during chemotherapy, ovarian transposition, embryo cryopreservation, oocyte vitrification, ovarian tissue cryopreservation, oocyte or embryo donation, and adoption for those who have already lost their ovarian reserve [32,33,34,35,36]. The efficacy, indications, possible risks, and future challenges of these options were critically discussed. Among these, medical therapy (particularly with gonadotropin-releasing hormone (GnRH) agonists) proved effective to protect against POI and preserve fertility in young women with ER-negative breast cancer undergoing chemotherapy, however, the use of GnRH agonists for fertility preservation remains controversial and GnRH agonists should not be relied on to preserve fertility [27,36]. Ovarian transposition has been suggested for women <40 years of age with low-grade cervical cancer before pelvic radiation because this safe and effective treatment could preserve ovarian function [36]. Oocyte and/or embryo vitrification and ovarian tissue cryopreservation are the two methods currently endorsed by the American Society for Reproductive Medicine. The choice of one technique over the other depends mostly on the age and pubertal status of the patient and personal and medical circumstances [37]. Embryo cryopreservation is a well-established fertility preservation technique used worldwide, and oocyte cryopreservation has also been an established and important component of fertility preservation. If some breast cancer patients cannot undergo ovarian stimulation before chemotherapy or have contraindications to exogenous gonadotropin, they can choose other methods. Ovarian tissue cryopreservation may be an option to preserve fertility in prepubertal girls and young women who require immediate chemotherapy. It has some advantages, especially for prepubertal patients: no need for ovarian stimulation, thus, no further risk for estrogen-sensitive cancer types, and preservation of more and better-quality primordial follicles of the ovarian cortex [38,39]. After 2018, there was a shift from hormone replacement therapy to more innovative treatment strategies. In vitro activation (IVA) of the ovarian cortex combined with autologous transplantation is one of the novel approaches to primary ovarian insufficiency and diminished ovarian reserve [40]. Later, a simplification of the technique designated “Drug-Free IVA” has shown encouraging results in patients with POI [41,42]. IVA represents a new frontier in the therapy of women with POI as well as patients with cancer desiring fertility preservation [43]. With the development of regenerative medicine and tissue engineering technology, mesenchymal stem cells (MSCs) have received increasing attention as a potential cell-based therapy for female infertility [44]. More and more relevant studies indicate that MSC transplantation, such as fetal mesenchymal stem cells from the liver [45], human embryonic stem cell-derived mesenchymal stem cells [46], and bone marrow mesenchymal stem cells [47], have some positive effects on the treatment of POI in animals. Although MSCs are not widely applied in clinical therapy because of the underlying molecular mechanism and safety, recent advances in MSCs therapy are likely to be translated to new therapeutic options bringing new hope to patients with POI [48]. In addition, mechanical disruption of the Hippo signaling pathway in combination with Akt stimulation [49], intraovarian injection of autologous platelet-rich plasma [49], antioxidant [50], acupuncture, [51], artificial ovary [52], etc., were among the latest hotspots in the last 5 years and they may represent promising treatments for the women with POI.

The top 10 publications with the highest citation in POI therapy are listed in Table 6. The impact factor (IF) of the top five journals were *Annals of Oncology* (IF = 32.98), *Lancet* (IF = 79.32), *New England Journal of Medicine* (IF = 91.24), *Human Reproduction Update* (IF = 15.61), and *Nature Medicine* (IF = 53.44), and all of the top five journals were classified in Q1 according to the 2020–2021 journal citation reports (JCRs). Furthermore, 80% of the top 10 journals in Table 6 belonged to Q1, indicating that the above journals had strong academic performance in the field of POI therapy. Based on the analysis of the top keywords in each cluster in combination with literature, the research hotspots in POI therapy were attributed as follows: (1) the indications, safe and effective treatment options, and risks of HRT for patients with POI [30,53,54]; (2) the application and development of the various fertility preservation strategies and assisted reproductive technologies for women with POI, especially in female tumor survivors with ovarian damage from chemotherapy [33,37,55,56]; (3) various new innovative experimental techniques and novel therapeutic strategies of fertility preservation in patients with POI [38,57].

## 5. Conclusions

The number of retrieved articles in the therapy for POI field has been rapidly growing, especially in the past 5 years, indicating an expanding interest in this topic. The most productive country, organizations, and journals were the USA, Shanghai Jiao Tong University, and *Human Reproduction*, respectively. The most commonly cited organization was Harvard University. The most influential journal and author were *Fertility and Sterility* and Nelson, L.M., respectively. Furthermore, the large number of keywords identified in seven research clusters showed the extensive scope of POI therapy. Therapy for POI mainly focused on the method for fertility preservation and the safe and effective treatment options of HRT for patients with POI. Various new innovative techniques and novel therapeutic strategies for fertility preservation are attracting increased attention, such as IVA of the ovarian cortex combined with autologous transplantation, BMSC transplantation, and intraovarian injection of autologous platelet-rich plasma, indicating the development potential of these therapies. The trends demonstrated in POI therapy may help researchers explore new directions for future research in this field. In summary, this study is the first bibliometric analysis to investigate a comprehensive overview of the therapy for POI research from 2000 to 2022. Researchers could clearly understand the fundamental knowledge structure including countries, institutions, authors, journals, keywords, and articles in the field of POI therapy from this bibliometric analysis. In addition, this study could also provide a valuable reference for researchers to understand the further diagnosis and therapy for POI.

The present study provided a deep insight into the status and trends of research on therapy for premature ovarian failure with the bibliometric analysis method. However, the search was limited to the Web of Science Core Collection indexed journals, and some documents that do not appear in the Web of Science Core Collection were missed. Only English articles were included, which may have decreased the number of articles. Despite these limitations, this paper still revealed the future research trends and hotspots in this research field of therapy of premature ovarian failure to some extent.

## Figures and Tables

**Table 1:** Top 10 most productive countries with publications on therapy for POI from 2000 to 2022.

Rank	Country	Documents	Citations	Total Link Strength
1	USA	297	12,909	861
2	China	230	3251	460
3	Italy	84	3053	267
4	France	55	1469	184
5	England	50	2588	297
6	Netherlands	44	1327	199
7	Canada	38	2000	200
8	Belgium	36	3900	181
9	Israel	31	2276	220
10	Scotland	27	1529	211

**Table 2:** Top 10 most productive organizations on therapy for POI from 2000 to 2022.

Rank	Organizations	Documents	Citations	Total Link Strength	Norm. Citations	Avg. Pub. Year
1	Shanghai Jiao Tong University	28	560	283	29.785	2018.0741
2	Nanjing Medical University	20	399	127	28.5468	2017.85
3	Universidade Estadual de Campinas	19	145	76	7.9928	2014.7368
4	National Institute of Child Health and Human Development	16	1198	183	20.1391	2006.3125
5	University of Edinburgh	16	1273	193	35.779	2012.25
6	Memorial Sloan Kettering Cancer Center	15	1648	222	34.1375	2012.8667
7	Harvard University	14	2001	43	49.6758	2009.8571
8	Shandong University	14	131	55	9.3923	2017.2857
9	Massachusetts General Hospital	13	850	68	28.4394	2011.7692
10	Monash University	13	257	69	11.5088	2013.4615

**Table 3:** Top 10 most productive publications on therapy for POI from 2000 to 2022.

Rank	Publication Titles	Documents	% of 1159	2020 Impact Factor
1	*Human Reproduction*	59	5.591	6.918
2	*Fertility and Sterility*	40	3.451	7.329
3	*Gynecological Endocrinology*	33	2.847	2.260
4	*Journal of Clinical Endocrinology Metabolism*	29	2.502	5.958
5	*Menopause-the Journal of the North American Menopause Society*	24	2.071	2.953
6	*PLoS ONE*	22	1.898	3.240
7	*Stem Cell Research Therapy*	21	1.812	6.832
8	*Reproductive Biomedicine Online*	18	1.553	3.828
9	*Climacteric*	17	1.467	3.005
10	*Reproductive Sciences*	16	1.381	3.060

**Table 4:** Top 10 core authors of publications on therapy for POI from 2000 to 2022.

Authors	Organizations	Documents	Citations	Avg. Pub. Year	Total Link Strength
Nelson, L.M.	Mary Elizabeth Conover Foundation, Inc. (USA)	18	1587	2010.14	40
Meirow, D.	Chaim Sheba Medical Center (ISRAEL)	3	913	2001.67	10
Sklar, C.A.	Memorial Sloan Kettering Cancer Center (USA)	8	852	2014.50	29
Kawamura, K.	International University of Health Advanced Reproduction Medical Research Center (JAPAN)	7	777	2016.71	27
Stovall, M.	University of Texas MD Anderson Cancer Center (USA)	3	735	2010.33	11
Suzuki, N.	Saint Marianna University (JAPAN)	5	658	2015.40	18
Sugishita, Y.	Yale University Laboratory Molecular Reproduction & Fertil Preservation (USA)	3	645	2014.00	17
Takae, S.	Saint Marianna University (JAPAN)	3	645	2014.00	17
Yoshioka, N.	Saint Marianna University (JAPAN)	3	645	2014.00	17
Ishizuka, B.	Rose Ladies Clinic, School of Medicine Tokyo (JAPAN)	5	575	2016.00	19

**Table 5:** Top 5 keywords in each cluster and top 10 keywords in each stage on therapy for POI from 2000 to 2022.

Cluster	Label	Occurrences	Period	Label	Occurrences
1	premature ovarian failure	381	2000–2009	hodgkins-disease	25
	hormone replacement therapy	108		testosterone	16
	menopause	106		luteinizing-hormone	16
	primary ovarian insufficiency	105		hypergonadotropic amenorrhea	15
	young-women	103		addisons-disease	11
2	expression	87		gonadotropin-releasing-hormone	10
	transplantation	86		menstrual-cycle	10
	granulosa-cells	65		agonist	10
	mice	64		precocious puberty	9
	apoptosis	63		androgens	9
	ovary	63		oophoritis	9
3	chemotherapy	176	2010–2017	premature ovarian failure	381
	fertility	143		women	227
	fertility preservation	137		failure	199
	anti-mullerian hormone	109		chemotherapy	176
	cancer	86		fertility	143
4	women	227		fertility preservation	137
	pregnancy	103		anti-mullerian hormone	124
	infertility	93		hormone replacement therapy	118
	in-vitro fertilization	53		menopause	106
	follicle-stimulating-hormone	39		primary ovarian insufficiency	105
5	failure	199	2018–2022	premature ovarian insufficiency	69
	premature ovarian insufficiency	69		mesenchymal stem-cells	24
	gene	43		dna-damage	12
	association	25		metaanalysis	9
	mutation	19		repair	9
6	therapy	72		variants	9
	systemic-lupus-erythematosus	33		acupuncture	9
	impact	23		cisplatin	8
	gonadotoxicity	20		antioxidant	8
	follow-up	17		rat model	7
7	cyclophosphamide	71		mtor	7
	reserve	48		in-vitro activation	7
	premenopausal women	35		autophagy	7
	breast cancer	31		platelet-rich plasma	7
	adjuvant chemotherapy	30			

**Table 6:** Top 10 highly cited documents on therapy for POI from 2000 to 2022.

Rank	Title	First Author	Journals	Publication Year	Total Citations
1	Tailoring therapies-improving the management of early breast cancer: st gallen international expert consensus on the primary therapy of early breast cancer	Coates, A.S.	*Annals of Oncology*	2015	1067
2	Livebirth after orthotopic transplantation of cryopreserved ovarian tissue	Donnez, J.	*Lancet*	2004	1054
3	Primary ovarian insufficiency	Nelson, L.M.	*New England Journal of Medicine*	2009	595
4	the effects of radiotherapy and chemotherapy on female reproduction	Meirow, D.	*Human Reproduction Update*	2001	541
5	Oocyte apoptosis is suppressed by disruption of the acid sphingomyelinase gene or by sphingosine-1-phosphate therapy	Morita, Y.	*Nature Medicine*	2000	459
6	The 2017 hormone therapy position statement of the north american menopause society	Pinkerton, J.V.	*Menopause-the Journal of The North American Menopause Society*	2017	424
7	Hippo signaling disruption and Akt stimulation of ovarian follicles for infertility treatment	Kawamura, K.	*Proceedings of the National Academy of Sciences of the United States*	2013	400
8	Predicting age of ovarian failure after radiation to a field that includes the ovaries	Wallace, W.H.B.	*International Journal of Radiation Oncology Biology Physics*	2005	363
9	Premature menopause in survivors of childhood cancer: a report from the childhood cancer survivor study	Sklar, C.A.	*Journal of the National Cancer Institute*	2006	353
10	Aromatic hydrocarbon receptor-driven bax gene expression is required for primary ovarian insufficiency caused by biohazardous environmental chemicals	Matikainen, T.	*Nature Genetics*	2001	344

## Data Availability

The datasets used and/or analyzed during the current study are available from the first author or the corresponding author upon reasonable request.
